# Translation and Validation of the Sinhala Version of the EORTC-QLQ-CR29 Questionnaire

**DOI:** 10.31557/APJCP.2020.21.1.31

**Published:** 2020

**Authors:** Dakshitha P Wickramasinghe, Praveen Dayasena, Sanjeewa Seneviratne, Dharmabandhu N Samarasekera

**Affiliations:** *Department of Surgery, Faculty of Medicine, University of Colombo, Sri Lanka. *

**Keywords:** Quality of life, colorectal cancer, sinhala

## Abstract

**Purpose:**

To validate the Sinhalese version of the EORTC QLQ-CR29 quality of life questionnaire for colorectal cancer.

**Method:**

We translated and pilot-tested (n=10) the questionnaire in Sri Lanka. We then assessed the reliability, factor structure and construct validity according to the EORTC guidelines. The testing was done in two tertiary care hospitals in Sri Lanka.

**Results:**

Of 110 participants, 103 (93%) returned the questionnaire, and 15 out of 20 (75%) returned the repeat-test questionnaire within a period of two weeks. Out of the original four scales three had better reliability than the original scales: urinary frequency (Cronbach α - 0.82), blood and mucus in stools (α-0.85), defaecation problems (α-0.76). The body image scale showed low reliability (α – 0.33). However, when one of the 3 items in the scale was omitted, it showed sufficient reliability (α – 0.74). Factor analysis showed good reliability for overall assessment of the two item scale for stool frequency (α – 0.82) and six item scale for defaecation problems (α – 0.76). Correlations between the subscales of CR29 and C30 questionnaires were below 0.40, except for body image, which correlated moderately (r-0.44) with emotional functioning. This confirmed satisfactory overall construct validity

**Conclusion:**

The scales for urinary frequency, blood and mucus in stools and defaecation problems which were reliable and had good validity. Body image scale failed to show sufficient reliability with the three-item scale and we suggest omitting one of the items to improve the overall reliability of the questionnaire. Construct validity was comparable to published data.

## Introduction

Colorectal cancer (CRC) is a seen in both developed as well as developing countries and accounts for 8% of all cancer related deaths worldwide. It is the 4th most common cause of death from cancer, with approximately 608,000 deaths annually (Ferlay et al., 2010). Among Sri Lankans in 2010, CRC was the 4th commonest cancer among males and 6th commonest cancer among females (Programme, 2016). Nearly a third will succumb to the disease by 2 years post-diagnosis but over 50% will be alive after 5 years. These values are comparable to Europe and the USA (Verdecchia et al., 2007). Disease survival, therefore, is not the sole goal and quality of life (QOL) plays an essential part in the treatment (Deshpande et al., 2011). No data regarding the QOL among CRC patients in Sri Lanka is available. 

Health related QOL measures (HRQOL) are multidimensional assessments. These include physical, psychological, cognitive functioning, emotional, sexual, spiritual and social domains (Osoba, 2011). This contrasts with evaluation of symptoms or the performance status, which are essentially uni-dimensional (Deshpande et al., 2011). There is considerable discordance between QOL measured by physicians and patients (Janse et al., 2004) and doctors have been found to underestimate the severity of symptoms (Stephens et al., 1997). Patient reported outcome measures (PROM) attempt to overcome this limitation. By definition, PRO is any data directly reported by the patient without an intermediary. This includes both family members and healthcare professionals (Willke et al., 2004). 

One of the most used PROM measures used in patients with cancer are the modular questionnaires developed by the European Organization for Research and Treatment of Cancer (EORTC). The EORTC QLQ-C30 (C30) is the questionnaire used in the initial assessment of the QOL in cancer patients. It has been translated and validated in over 90 languages and has been used in over 3,000 studies throughout the world (Cancer, 2016a). It is supplemented by disease-specific modules, e.g.- colorectal (C29), breast (BR23) (Cancer, 2016b). 

Despite having a literacy rate of 91.2% in 2012 (UNICEF, 2013), available data suggest that the English literacy rate in Sri Lanka was less than 25% in 2001 (Little and Hettige, 2014). By definition, PROMs are documented by the patient. Assistance in completing the questionnaires is not permitted. The EORTC QLQ-C30 is available in Sinhala. However, the EORTC QLQ-C29 which focusses on CRC is not available in Sinhala. The objective of this study was to translate and psychometrically validate the CR29 questionnaire in a cohort of CRC patients in Sri Lanka. 

## Materials and Methods

*Translation procedure*

The CR29 questionnaire was translated according to the guidelines of EORTC (Kuliś et al., 2016). The forward translation was done by the Department of Sinhala while the back-translation was done by the Department of English, Faculty of Arts, University of Colombo. 

The questionnaire was pilot tested on 15 patients who have had surgery for CRC at least 3 months ago. The content was assessed for difficulties in understanding or answering, confusing or offensive (Kuliś et al., 2016), and the questionnaire was amended accordingly. The approval of the EORTC team was obtained at each step.

*Participants*

*The study was conducted as a descriptive study*

The participants were recruited from the National Hospital of Sri Lanka and the National Cancer Institute of Sri Lanka, from January – October 2017. Any adult (older than 18 years) patient who had been treated for histologically confirmed CRC and could read Sinhala was eligible to be recruited. Patients being treated for other malignancy (excluding metastatic disease of CRC) were excluded. 

Patients were managed according to present CRC management guidelines. 

*Measures*

The C30 is the core questionnaire and comprises the following components. 

• Five functional dimensions - physical functioning, role functioning, cognitive functioning, emotional functioning and social functioning.

• Three symptom scales - fatigue, pain, and nausea/ vomiting, and 6 individual symptoms.

• One question each for global health-related quality of life and financial impact

All components except the QOL are marked on a 4 item Likert scale. The validity of the Sinhala version of the C30 questionnaire has been established in Sri Lanka (Jayasekara et al., 2008). 

The CR29 questionnaire was designed by EORTC to supplement the C30 in assessing CRC patients. The original (Whistance et al., 2009) as well as the translated versions (Ihn et al., 2015; Magaji et al., 2015; Lin et al., 2017) have been used extensively. The CR29 is comprised of 5 functional scales – Body image, anxiety, weight, and sexual interest in men and women, and 18 symptom scales. There were separate questions for patient with / without a stoma and male / female. Most questions request the patient to reflect on their symptoms over the past week. The questions pertaining to the sexuality require the patients to evaluate the past 4 weeks.

The scores for all questions are converted to a scale with a range from 0 to 100. 

*Data collection procedure*

All participants completed the C30 and CR29 questionnaires at the same visit. The study was explained to the participants and the return of a completed questionnaire was considered implied consent. Some patients were invited to complete the questionnaire for the 2nd time, to evaluate the test-retest reliability.

The data on their demographics, education, financial status and overall health were collected using a proforma. Details of the tumour and treatment was obtained by reviewing the patient records. 

*Statistical analysis*

The aspects assessed include the reliability, and convergent, divergent and known-groups validity. The internal consistency of the scales was assessed using the Cronbach’s alpha test. Using multi-trait scaling analysis, we looked for hypothetical multi-item scales that the original items from the questionnaire would fit into. We expected to see a correlation > 0.4. A good discriminant validity was confirmed if the correlation between the item and its own scale was higher than the item with other scales.

Convergent and divergent validity were evaluated using correlations between items of the C30 and CR29 questionnaires. We expected items that were conceptually related (e.g. physical functioning and fatigue) to better correlate (Pearson rho >0.5) than areas that were not related (Pearson rho <0.2) (Arraras et al., 2011). Reliability was tested using Interclass Correlation Coefficient (ICC) and interpreted according to accepted standards (Cicchetti, 1994).

Statistical analysis was performed using Statistical Package for Social Studies (SPSS) version 20 (IBM Corp. Released 2011. Armonk, NY: IBM Corp.). 

*Ethical approval*

Ethical approval was obtained from the ethics review committees of the respective hospitals. 

## Results

Of 110 participants, 103 (93%) returned the questionnaire. Five of the seven participants who did not complete the questionnaire could not read the lettering because they did not use spectacles. The remaining had returned incomplete questionnaires. The sociodemographic data of the participants are shown in Table 1.

Most participants were able to complete both questionnaires in less than 30 minutes. None required clarification of statements. There was a total of 36 (1.2%) and 60 (2%) missing answers in the returned CR30 and CR29 questionnaires, respectively. 

The mean age of the population was 57.2 (± 12) years. Median Charlson comorbidity index was 2 (± 1.6) and the median Karnofsky performance scale was 80.2 (± 16.9). 

The scores of the CR29 questionnaire, with the percentage values of respondents in the lowest and highest scores are shown in Table 2.

*Internal consistency*

Out of the four scales, three had better reliability than the original publication (Whistance et al., 2009) (urinary frequency – Cronbach α - 0.82 vs original α - 0.75, blood and mucus in stools α - 0.85 vs original α - 0.69 and defaecation problems α - 0.76 vs original α – 0.70). The body image scale showed low reliability (α – 0.33) compared to the original (α – 0.84). However, when one of the 3 items in the scale was omitted, it showed sufficient reliability (α – 0.74).

*Validity*

Several scales of C30 and CR29 had correlations over 0.5. These include pain and appetite loss (0.548), trouble with taste and appetite loss (0.541), body image and emotional functioning scale (0.538) and pain and insomnia (0.524). Other scales that showed high correlations include abdominal pain and buttock pain (r=0.454), abdominal pain and insomnia (r=0.454), buttock pain and anxiety (r=0.413), dry mouth and trouble with taste (r=0.456), dry mouth and pain (r=0.467), dry mouth and appetite loss (r=0.453), trouble with taste and pain (r=0.5), pain and diarrhoea (r=0.486). All other scales had low correlations. 

Factor analysis showed good reliability for overall assessment of the two-item scale for stool frequency (α – 0.82) and six item scales for defaecation problems (α – 0.76). Patients with a stoma had a higher reliability in the two-item stool frequency scale (α – 0.90), when compared to non-stoma patients (α – 0.79). 

The variance extracted was higher than the square of the correlation for all analysis. Therefore, divergent validity was established. 

*Known group validity*

The scores obtained by clinically distinct groups for the scales and single items are shown in Table 3. Most scales and items showed the expected differences. However, statistically significant differences were seen in only a few items between patients who have / didn’t have stomas, and the different surgeries. The most significant changes were seen in patients of Karnofsky score less / more than 80%.

*Reproducibility*

Fifteen out of 20 (75%) returned the repeat-test questionnaire within a period of two weeks (without significant events affecting quality of life). Body image (ICC 0.796), defaecation / stoma related problems (0.764), urinary incontinence (0.784), abdominal pain (0.803), Hair loss (0.777) and anxiety (0.769) had excellent reproducibility. Buttock pain (0.739) had a good reproducibility while the other scales had fair reproducibility.

**Table 1:** Socio Demographic and Clinical Details of the Participants

Variable	N (%)
Sex	
Male	53 (51.5)
Female	50 (48.5)
Religion	
Buddhist	81 (78.6)
Christian and catholic	15 (14.6)
Islam	5 (4.8)
Other	2 (1.9)
Level of education	
University	9 (8.7)
Completed AL	23 (22.3)
Completed OL	41 (39.8)
Less than OL	28 (27.2)
No formal education	2 (1.9)
Tumour location	
Colon	24 (23.3)
Rectum	79 (76.7)
Stoma	
Yes	37 (35.9)
No	66 (64.1)

**Table 2:** Quality of Life Scores According to EORTC-QOL-CR29 Questionnaire

	Items	Mean (± SD)	Alpha	Percentage of patients with the lowest scores	Percentage of patients with the highest scores	Range
Item name / scale						
Urinary frequency	1.2	48.9 ± 26.6	0.82	6.1	10.1	0-100
Blood and mucus in stools	8.9	16.5 ± 26.4	0.85	59.8	2.9	0-100
Body image	15-17	24.9 ± 24.3	0.33 / 0.74 *	26	2.1	0-100
Defaecation / stoma related problems	19-24	22.5 ± 19.3	0.76	9.5	1.1	0-100
Urinary incontinence	3	7.7 ± 20.5		84	1	0-100
Dysuria	4	15.7 ± 25.3		65	4	0-100
Abdominal pain	5	27 ± 35.0		55	11	0-100
Buttock pain	6	30.3 ± 34.9		47	12	0-100
Bloated Feeling	7	6.4 ± 17.6		85.9	1	0-100
Dry mouth	10	25.0 ± 29.3		49	5	0-100
Hair loss	11	32.6 ± 38.9		51	17.3	0-100
Trouble with Taste	12	28.0 ± 34.4		52	10	0-100
Anxiety	13	34.0 ± 33.1		36	12	0-100
Weight	14	15.5 ± 23.9		63.6	3	0-100
Patients without stoma						
Flatulence	19	40.9 ± 32.9		23	16.4	0-100
Faecal incontinence	20	16.1 ± 28.1		69.4	4.8	0-100
Sore skin around anus	21	23.3 ± 28.4		50.8	4.8	0-100
Stool frequency	22.23	21.7 ± 26.8		42.9	3.2	0-100
Embarrassed by bowel movement	24	11.5 ± 25.7		78.7	4.9	0-100
Defaecation problems	19-24	23.1 ± 21.4		11.9	1.7	0-100
Patients with a stoma (n=35)						
Flatulence	19	23.8 ± 23.7		42.9	14.3	0-66.7
Faecal incontinence	20	13.5 ± 21.5		67.6	8.1	0-66.7
Sore skin around stoma	21	21.6 ± 23.8		45.9	2.7	0-100
Stool frequency	22.23	18.9 ± 24.6		54.1	2.7	0-83.3
Embarrassed by stoma	24	28.8 ± 32.5		43.2	10.8	0-100
Stoma related problems	25	20.7 ± 25.3		54.1	13.5	0-66.7
Stoma care problems	19-24	21.1 ± 15.5		5.7	5.7	0-61.1
Male						
Sexual function in men	26	18.0 ± 25.4		60	2	0-100
Impotence	27	30.5 ± 37.9		51.1	17	0-100
Female						
Sexual function in women	28	3.6 ± 10.5		89.1	10.9	0-33.3
Dyspareunia	29	5.6 ± 16.3		88.1	4.8	0-66.7

*, Modified body imaging score

**Table 3:** Comparison of QOL Data between Known Groups

	Stoma			Type of surgery					Karnofsky score		
	Yes	No		AR	APR	AR vs APR	Colectomy	AR vs APR vs Colectomy	< 80%	>80%	
	Mean ± SD	Mean ± SD	p	Mean ± SD	Mean ± SD	p	Mean ± SD	p	Mean ± SD	Mean ± SD	p
Blood and mucus stools	13.43 ± 26.37	18.25 ± 26.39	0.33	13.96 ± 25.00	2.38 ± 6.05	0.11	4.55 ± 11.71	0.13	22.22 ± 27.53	13.64 ± 25.47	0.06
Urinary frequency	50.93 ± 31.10	48.41 ± 25.35	0.8	40.09 ± 25.90	55.13 ± 36.88	0.35	51.45 ± 21.27	0.13	53.13 ± 24.84	47.01 ± 27.36	0.39
Body image	33.64 ± 27.15	19.63 ± 21.10	0.01	27.16 ± 23.22	29.91 ± 31.55	0.97	19.32 ± 25.56	0.24	39.26 ± 27.33	18.35 ± 19.92	0
Stoma related problems	-	-	-	24.38 ± 20.93	15.08 ± 10.32	0.25	12.70 ± 10.71	0.07	30.29 ± 19.66	18.75 ± 18.18	0
Urinary incontinence	15.32 ± 30.02	3.17 ± 9.86	0.02	6.31 ± 18.98	23.81 ± 37.96	0.08	2.90 ± 9.60	0.08	12.12 ± 23.30	3.48 ± 10.27	0.03
Dysuria	20.72 ± 31.77	14.81 ± 25.24	0.6	11.71 ± 17.94	28.57 ± 43.08	0.64	13.04 ± 31.36	0.51	22.22 ± 28.46	12.44 ± 23.09	0.05
Abdominal pain	26.13 ± 36.12	39.15 ± 91.88	0.39	19.82 ± 28.82	35.71 ± 44.27	0.64	30.43 ± 38.81	0.7	31.31 ± 36.27	24.88 ± 34.50	0.35
Buttock pain	24.32 ± 32.06	33.86 ± 36.17	0.2	24.32 ± 27.94	30.95 ± 38.04	0.77	18.84 ± 33.07	0.36	38.38 ± 33.46	26.37 ± 35.07	0.05
Bloated feeling	6.31 ± 18.98	6.45 ± 16.90	0.88	1.80 ± 7.64	4.76 ± 12.10	0.3	2.90 ± 9.60	0.58	8.33 ± 18.93	5.47 ± 17.01	0.36
Dry mouth	25.2 ± 29.8	24.9 ± 29.3	0.96	27.9 ± 30.9	11.9 ± 16.6	0.11	24.6 ± 33.7	0.29	35.35 ± 31.11	19.90 ± 27.25	0.01
Hair loss	39.64 ± 41.45	28.42 ± 36.94	0.17	35.14 ± 37.64	33.33 ± 45.29	0.69	42.03 ± 41.70	0.78	27.27 ± 36.76	35.38 ± 39.91	0.4
Trouble with Taste	26.13 ± 35.25	29.10 ± 34.13	0.57	19.82 ± 28.82	23.81 ± 37.96	0.93	36.23 ± 38.81	0.23	35.35 ± 37.21	24.38 ± 32.61	0.1
Stool frequency	18.92 ± 24.58	21.69 ± 26.88	0.51	23.68 ± 25.30	13.10 ± 22.81	0.074	6.52 ± 13.05	0	27.27 ± 26.62	17.69 ± 25.49	0.04
Anxiety	31.53 ± 32.34	35.45 ± 33.80	0.58	28.83 ± 28.50	35.71 ± 38.04	0.72	33.33 ± 37.61	0.93	46.46 ± 33.27	27.86 ± 31.57	0.01
Weight	21.30 ± 27.78	12.17 ± 21.00	0.08	14.41 ± 21.57	17.95 ± 22.01	0.55	8.70 ± 22.96	0.17	24.24 ± 27.98	11.11 ± 20.53	0.01
Flatulence	23.8 ± 23.6	41.0 ± 33.0	0.02	27.7 ± 27.4	21.4 ± 21.1	0.55	40.9 ± 32.4	0.15	43.75 ± 32.17	30.21 ± 29.53	0.03
Faecal incontinence / leakage from stoma	13.5 ± 21.4	16.4 ± 28.3	0.93	19.4 ± 25.7	7.1 ± 14.2	0.11	7.2 ± 19.9	0.04	19.19 ± 27.68	13.33 ±24.86	0.17
Sore skin	21.62 ± 23.85	23.28 ± 28.48	0.89	22.81 ± 23.39	9.52 ± 15.63	0.03	10.14 ± 18.63	0.01	27.27 ± 25.62	21.03 ± 27.37	0.15
Embarrassed by bowel movement	26.13 ± 47.23	26.67 ± 0.00	0	14.71 ± 12.07	19.05 ± 25.20	0.33	22.53 ± 96.30	0.02	35.42 ± 38.74	9.90 ± 19.41	0
Stoma care problems	-	-	-	28.1 ± 18.2	15.1 ± 10.3	0.2	13.9 ± 11.8	0.1	30.29 ± 19.66	18.43 ± 18.15	0
Male sexual function	15.87 ± 22.65	19.05 ± 27.86	0.83	21.05 ± 25.36	10.00 ± 16.10	0.27	29.17 ± 37.53	0.44	12.28 ± 19.91	21.51 ± 27.95	0.27
Impotence	43.33 ± 40.61	21.79 ± 33.92	0.05	28.07 ± 35.60	51.85 ± 47.47	0.18	8.33 ± 15.43	0.1	43.14 ± 42.11	23.33 ± 34.07	0.08
Female sexual function	0	5.38 ± 12.46	0.12	3.51 ± 10.51	0	0.51	4.44 ± 11.73	0.75	0	4.50 ± 11.55	0.25
Dyspareunia	3.03 ± 10.05	6.67 ± 18.36	0.68	9.80 ± 22.87	0	0.44	2.38 ± 8.91	0.51	14.29 ± 26.23	3.81 ± 13.46	0.13

## Discussion

This is the first study focussing on the psychometric properties of the Sinhala version of the CR29 questionnaire. The results indicate internal consistency, validity and reproducibility comparable to the original study (Whistance et al., 2009) as well as other translated versions (Arraras et al., 2011; Ihn et al., 2015; Magaji et al., 2015; Lin et al., 2017). The questionnaire also has good discriminant power for identification of differences in QOL between conceptually different patient populations. The scales and item scores were found to be independent of those on C30 questionnaire. 

The time taken for the participants to complete the questionnaires was comparable to other studies (Ihn et al., 2015). Also, we saw very few missing data. Both these suggest that the questionnaires are easy to understand and complete, not burdensome and therefore produce high compliance. 

Three of the 4 scales in the questionnaire showed better internal consistency than the original article (Whistance et al., 2009) and comparable results to other translated versions (Ihn et al., 2015; Magaji et al., 2015; Stiggelbout et al., 2016; Lin et al., 2017). However, the body image scale had a low internal consistency when all 3 questions were included. When only questions 45 and 46 were included, the internal consistency improved to .739 which was comparable to other studies. The excluded question focusses on dissatisfaction about the body. Previous studies have shown that the Asian patients adapt well post surgically. Even the presence of a stoma doesn’t affect their QOL (Hamashima, 2002). Therefore, it is possible that Sri Lankan patients do not have significant dissatisfaction of their body post-surgically. We would like to encourage future users of the questionnaire to be cognizant of this variation in their interpretation of QOL data with the Sinhala version of the CR29. 

Except for a few scales and items highlighted above, all other items and scales of the 2 scales had correlations less than .04. These results confirm independence in these items, similar to other translated questionnaires (Ihn et al., 2015; Lin et al., 2017).

There is conflicting evidence on whether the presence of a stoma impairs (Liao and Qin, 2014) or doesn’t affect (Orsini et al., 2013) the quality of life of CRC patients. Our findings showed a statistically significant difference in only body image and urinary incontinence, both being worse with a stoma. Similar to the above observation where our participants had less dissatisfaction, we believe Sri Lankan patients adapt better to the presence of a stoma. However, we saw significant differences in multiple scales and items in patients with poor functional status (Karnofsky score <80% vs >80%). Patients with a higher Karnofsky scores consistently had better scores for functional scales. These observations have been made by previous researchers as well (Lin et al., 2017). It is possible that patients with a lower performance status have lower expectations and therefore are less affected by post treatment changes in life-style. 

We saw good to excellent reproducibility in many of the scales while some had fair reproducibility. We feel the lower reliability on these scales is mainly due to the small size of the retest population than due to the stability of the questionnaire. 

The study had several limitations. It recruited participants from only 2 centres. However, they are the biggest centres managing patients with malignancies in Sri Lanka and most patients in the country would have been treated there. Therefore, we feel we had a representative population. Our re-test group was small and would have contributed to the lower reproducibility results in some scales.

In conclusion, the Sinhala version of the EORTC-QOL-CR29 questionnaire is a valid and reliable tool in assessing quality of life in patients with colorectal cancer. It would benefit clinicians and researchers alike. 
